
JOURNAL INFORMATION
==============================
NLM Title Abbreviation: ScientificWorldJournal
Journal ID: ScientificWorldJournal
NLM Journal ID: 101131163
Journal ID: TSWJ

The Scientific World Journal

ISSN: 2356-6140
EISSN: 1537-744X
Publisher: Wiley

ARTICLE INFORMATION
==============================
PMCID: PMC6083839
DOI: 10.1100/tsw.2001.411
Article ID: 250383
Article version: 1
Subjects: Extended Abstract

CGRP in Primary Headaches

Edvinsson Lars
Department of Internal Medicine, University Hospital, 221 85 Lund, Sweden
Publication date: 2001
Electronic publication date: 2001 Nov 8
Volume: 1
First page: 29
Last page: 29
Copyright: Copyright © 2001 Lars Edvinsson.
Copyright year: 2001
License: This is an open access article distributed under the Creative Commons Attribution License, which permits unrestricted use, distribution, and reproduction in any medium, provided the original work is properly cited.
License URL: https://creativecommons.org/licenses/by/3.0/

==============================
4th International Meeting on Calcitonin Gene-Related Peptide (CGRP)
*TheScientificWorld* (2001) 1(S1), 29
ISSN 1532-2246; DOI 10.1100/tsw.2001.411

### CGRP IN PRIMARY HEADACHES

Lars Edvinsson
Department of Internal Medicine, University Hospital, 221 85 Lund, Sweden

Calcitonin-gene related peptide (CGRP)-containing nerve fibres supply vessels of different locations, both peripheral and cerebral. The intracranial vascular bed is supplied by CGRP fibers that originate in the trigeminal ganglion. When these fibres are activated they release CGRP as seen both in man and animals. CGRP initiates relaxant responses through an interaction with G-protein coupled receptors that activate adenylyl cyclase. CGRP mediates relaxation via two functional receptors subtypes, the $CGRP_1$ and the $CGRP_2$ receptors. The former of the CGRP receptors has been characterized by the use of the antagonist $CGRP_{8-37}$, compound 1 and BIBN4096BS as well as by molecular methods.

In migraine with or without aura, and in cluster headache there is a marked release of CGRP in parallel with the headache. Treatment of the patients with a triptan relieves the pain and the CGRP levels normalize, probably via a presynaptic effect. In cluster headache and in chronic paroxysmal hemicrania there is in addition release of VIP, from parasympathetic nerves. These clinical data strongly advocates the use of a CGRP blocker in the treatment of acute attacks of primary headaches.
